# Event and Comment

**Published:** 1922-06

**Authors:** 


### DENTAL REGISTER

THE MAGAZINE OF DENTISTRY

Vol. LXXVI JUNE, 1922 No. 6

### EVENT AND COMMENT

**Balance in Dental Education** In these days when so much is being said about improving our system of training the future dentist we sometimes wonder if we are making real progress, or doing more for humanity in our endeavors to extend the time over which it is proposed to extend the course of instruction.

There are so many factors involved in a study of this problem that it seems difficult to specify any one factor in particular, without throwing the general balance of the equation out of harmonious working proportions. The economic factors, of course, control the essential basis for any readjustment that may be considered either advisable or practicable; and there is no known mathematical formula that will serve adequately for the adjustment of such a problem. The old and tried methods of "fitting and trying" are the tedious processes of evolution that have worked so well that they have become standardized by mere traditions. We now seem to be approaching a time when the wit and wisdom of the present should be utilized as never before to formulate a better system of handling so important a matter with a greater degree of scientific accuracy than it was formerly believed practicable. If it were practicable to begin the study of this matter mathematically, for instance, how can we go about making preliminary measurements, or to even visualize and logically conceive it? Can it be measured cubically or spheroidally, or is it polyhedral in form, or just a mere mass? In the mass it may be stable, but it is imponderable and inartistic, unless the cone with its wide base and pointed apex, like the Egyptian pyramids, have both qualities. Sir Michael Foster likened true education to that form in his famous analogy. Our trouble seems to start with a faulty conception, or of a base lacking completeness. Shall we liken our conception of human needs to our artistic requirements to meet our present stage of development? We have developed our practice and theories concurrently, and our ideals are the result of much conformity to man-made patterns, and like "Topsy, we don't know who fathered us, we just grew," without design or mathematical precision. In spite of these natural controls we have accomplished a tremendous uplift for humanity. It is true we have made many mistakes in the building of our educational systems, and none are yet running true to form. We have already torn down many faulty edifices and done a great deal of remodeling. Our ideals have suffered in a similar way, some which we thought fundamental have by the lapse of time and experience, we find been subjects of radical reformations, until there seems at times no hope for permanent dogmatism. On the contrary, we seem quite ripe for the inauguration of a new era of attacking the problem from a new angle that promises more light on a problem of such vital interest.

Our natural habit of caution, however, steadies us when tempted to speculate with so far-reaching a problem, and we hold back because all interested lack foresight and wisdom.

Accumulated knowledge and clinical experience urges some to dare the adoption of the newer conceptions of more recent clinical practice. In general, we believe to act hastily means probable disaster, but fortified with well-grounded scientific study we are beginning to risk some procedures not warranted by traditional experience.

On the whole, we are steadily moving forward with a better degree of confidence and a spirit of hope, because the needs for our profession keep up an increasing demand. For us today the world's greatest need is better dental hygiene. The whole range of better scientific medical art is making a mighty appeal in every department of dentistry, to take care of the apparent needs to relieve disease by this form of service.

While it is very important that dental medicine shall be fostered in every proper way, we should not at any time fail to realize in our endeavors that some of our cherished ambitions are at present impracticable. In our attempts to realize our ideals some rational efforts may be inadvisable because at present they may be impossible to attain with successful results.

If it can be definitely determined that a five-year course of instruction will meet the greatest needs to the greatest numbers, the course of study should be advanced to the limit, in spite of possible obstacles. We want the acme of dental health, and we must work for this ideal in our standards of education with all practicable speed that will insure a balanced equilibrium of efficiency.

